# Exploring Engagement with the Online ‘Parenting with Anxiety’ Intervention Within a Community Setting: A Quasi-Experimental Feasibility Study

**DOI:** 10.3390/bs16071149

**Published:** 2026-07-08

**Authors:** Mia Carter, Hannah Frith, Sam Cartwright-Hatton, Abby Dunn

**Affiliations:** 1 Department of Psychology, University of Surrey, Guildford GU2 7XH, UK; 2 Department of Psychology, University of Sussex, Brighton BN1 9QH, UK

**Keywords:** anxiety, parent anxiety, child anxiety, digital mental health intervention (DMHI), parenting, online engagement, therapist support

## Abstract

Background: Parental anxiety is a significant risk factor for child anxiety, yet evidence-based support remains inaccessible to most. The predecessor ‘Parenting with Anxiety’ (PWA) RCT evaluated an unguided online intervention across 1800 parents and demonstrated effectiveness but reached a homogeneous sample with only 19% completing all modules. Objective: To explore whether community embedding and brief therapist support could broaden reach and improve engagement. Method: A quasi-experimental design of 28 eligible parents (96% female; 46% from minoritised ethnic backgrounds; 50% experiencing financial hardship) were self-allocated to therapist-support (*n* = 13) or self-guided (*n* = 15) conditions. Primary outcomes were acceptability and engagement (logins; modules accessed and completed). Parental and child anxiety were assessed at baseline, post-intervention, and six-month follow-up using paired measures (*n* = 11). Results: In total, 80% of self-guided participants never logged in versus 31% of therapist-support participants; as arms differed in recruitment pathway, this cannot be attributed to therapist support alone. Therapist-support parents reported high satisfaction and parental anxiety improved reliably in four of 11 participants at post-intervention and four at follow-up, with none reliably deteriorating. Child anxiety showed no consistent pattern. Conclusions: Community-embedded, therapist-supported PWA delivery was feasible, acceptable, and associated with higher engagement, though self-selection and the small sample preclude causal interpretation.

## 1. Introduction

Anxiety disorders are highly prevalent and represent a significant public health concern (Fineberg et al., 2013; Newlove-Delgado et al., 2022; NHS England, 2023; Santomauro et al., 2021). In the UK, an estimated 8.2 million adults experience a probable anxiety disorder (Santomauro et al., 2021), and a substantial proportion of these adults are parents (Cole et al., 2021). Anxiety is also the most common mental health difficulty among children and adolescents (Polanczyk et al., 2015); around one in six children meet the criteria of an emotional disorder (Newlove-Delgado et al., 2022), which often persist into adulthood (Last et al., 1997), are commonly left untreated (Wang et al., 2005), and can contribute to further emotional and functional difficulties over time (Bittner et al., 2007). Identifying effective, accessible, and preventative approaches therefore remains an important public health priority.

Parental anxiety is a well-established risk factor for the development of anxiety disorders in children (Hudson & Dodd, 2012), reflecting a combination of genetic, environmental, and contextual influences rather than a single causal pathway. A body of evidence implicates a range of anxiogenic parenting behaviours as possible contributors to this intergenerational transmission. For example, parents may, often unintentionally and with protective intentions, model heightened fear expression, convey threat-related information, encourage avoidance of feared situations, or struggle with consistent and appropriate behaviour management or autonomy promotion (Murray et al., 2009; de Rosnay et al., 2006; Robinson & Cartwright-Hatton, 2008; Hudson et al., 2009; Thirlwall & Creswell, 2010). Environmental influences on the development of child anxiety have been shown to be more influential than genetic factors (Eley et al., 2015), suggesting that these behaviours, while not deterministic, represent meaningful and modifiable targets for preventative interventions aimed at reducing children’s anxiety risk.

Parent-led cognitive behavioural therapy (CBT)-based interventions have shown positive outcomes in reducing child anxiety (Lawrence et al., 2017; Ginsburg et al., 2015; Cartwright-Hatton et al., 2011; Bennett et al., 2016; James et al., 2020). Most research targets children who already meet diagnostic thresholds or show clear symptoms of anxiety. In contrast, preventative approaches seek to reduce the likelihood of anxiety developing in children who may not yet present with difficulties but are considered at risk because of constitutional, environmental, or parental factors. Within this preventive context, the Raising Confident Children (RCC) programme was originally designed as a face-to-face group intervention for highly anxious parents. The RCC intervention is grounded in CBT and social learning theory, targeting key anxiogenic parenting behaviours (e.g., avoidance, over-reassurance, overprotection, and modelling of fear) and behavioural parenting principles (e.g., positive reinforcement, limit-setting, anxiety exposure). In a randomised controlled trial (RCT), children whose parents completed the in-person programme were 16.5% less likely to develop an anxiety disorder one year later compared to those in the control group (Cartwright-Hatton et al., 2018), highlighting the potential of parenting-based preventative approaches.

However, access to in-person parenting programmes is restricted by barriers, such as time, childcare, costs, stigma, and limited service availability (Reardon et al., 2020; Signorini et al., 2018). As a result, many anxious parents do not receive evidence-based support (Alonso et al., 2018). This has led to growing interest in digital mental health interventions (DMHIs) to improve access. DMHIs can reduce logistical and psychological barriers, expand reach, and reduce reliance on limited clinical resources (Canário et al., 2022; Kazdin, 2019). Systematic reviews have demonstrated that digital delivery of evidence-based parenting programmes is feasible, with completion rates ranging from 41 to 99%; where behavioural outcomes were measured (*n* = 4), moderate effect sizes were reported for both parent and child outcomes (0.61 and 0.46, respectively, Cohen’s *d*) (Breitenstein et al., 2014). Furthermore, digital adaptations of parenting programmes, such as the Triple P [Positive Parenting Programme] Digital, have shown promising feasibility and acceptability (Ehrensaft et al., 2016). With most adults in the UK having reliable internet access (Office for National Statistics, 2018), digital delivery provides a potentially scalable route for offering early intervention and bridging gaps in service provision.

The ‘Parenting with Anxiety’ (PWA) project adapted the face-to-face RCC course into an online, unguided, and modular intervention rooted in the same cognitive behavioural and social learning principles designed to help parents develop a calm, consistent approach to managing their child’s behaviour while reducing anxiogenic parenting responses, such as avoidance, over-reassurance, and overprotection. In the trial evaluating this adaptation, the course comprised one core introductory module completed by all participants, followed by a further eight modules covering topics, including reducing avoidance with exposure; using play to build children’s confidence; emotion coaching; managing difficult behaviours through positive reinforcement, consequences, and limit-setting; reducing parental overprotection and modelling confident behaviour; and the role of sleep, diet, and exercise in children’s mental health. Each participant was randomly allocated to seven of these eight additional modules to permit a planned analysis of individual module effects. Each module took approximately 20–30 min to complete and combined video and animated content with written material, case examples, quizzes, and action-planning homework tasks, with no clinical input provided at any point beyond optional technical support. PWA was evaluated in a large RCT involving over 1800 highly anxious parents (Dunn et al., 2024). Small but significant reductions in both child and parent anxiety were found, with effects maintained for up to two years. Engagement was highly variable, as only 19% of participants completed all modules, with higher engagement associated with better outcomes. The sample was also demographically homogenous (predominantly white, well-educated, and financially stable), raising concerns about generalisability and potential digital exclusion. These findings suggest that although unguided digital interventions can be effective, additional approaches may be required to improve engagement and to broaden reach to more diverse populations.

Despite their promise, DMHIs face challenges with real-world engagement; attrition is high and usage typically declines sharply over time, with most users disengaging within the first weeks of access (Baumel et al., 2019; Wasil et al., 2020). The generalisability of results beyond controlled trial settings is also poorly understood. Engagement difficulties can limit both effectiveness and scalability, particularly among families who experience barriers to digital access or face high levels of social adversity (Hollis et al., 2017). Therapist-support or hybrid DMHIs may enhance motivation, accountability and engagement, with multiple reviews finding that human support can improve engagement and impact of online interventions (Werntz et al., 2023; Andersson et al., 2019; Philippe et al., 2022; Baumeister et al., 2014). However, findings are mixed, with some studies reporting limited added benefit compared with self-guided delivery (Gardner et al., 2022; Andrews et al., 2023).

Building on recommendations from the predecessor trial (Dunn et al., 2024), this study explored whether brief therapist support was associated with differences in engagement with the online PWA intervention and assessed the feasibility of delivery through an existing community-based service. The therapist-support arm was informed by evidence that human support in DMHIs can strengthen motivation, accountability, and persistence, with approaches such as supportive accountability, collaborative goal-setting, and personalised guidance shown to enhance engagement (Mohr et al., 2011; Yardley et al., 2016; Werntz et al., 2023). To address concerns that DMHIs may exacerbate inequalities without an inclusive design, the study was conducted in collaboration with Home-Start, a UK charity supporting families facing social, financial, and emotional challenges, to facilitate participation from a more diverse group of parents. This exploratory feasibility study addresses two research questions: (1) how does engagement with the online PWA intervention differ between parents receiving brief therapist support and those using it in a self-guided format and what factors might account for any difference observed? And (2) is it feasible and acceptable to deliver PWA within a community-based setting (Home-Start)?

## 2. Materials and Methods

### 2.1. Study Design

This quasi-experimental, non-randomised study evaluated the feasibility and acceptability of the PWA online intervention within two community-based Home-Start branches. Participants were self-allocated to either a therapist-support or a self-guided arm to reflect preference rather than random assignment. All parents were asked to complete the online intervention within eight weeks. The study extended the predecessor trial (Dunn et al., 2024) by examining engagement and delivery embedded within a community-based context. Reporting followed Transparent Reporting of Evaluations with Non-randomised Designs (TREND) guidelines (Des Jarlais et al., 2004). Ethical approval was granted by the Faculty of Health and Medical Sciences’ University Ethics Committee (UEC), University of Surrey (protocol code, FHMS 23–24 190 EGA; date of approval, 28 October 2024). This study was not preregistered.

### 2.2. Setting

The study was conducted across two Home-Start branches in Surrey, UK. Home-Start is a voluntary-sector charity offering practical and emotional support to families with children under five through home-visiting volunteers and group-based activities. The Guildford and Elmbridge branches take referrals to support families experiencing a range of challenges, including social adversity, financial strain, mental and physical health difficulties, disability, housing problems, and social isolation. Conducting the study in this setting aimed to engage parents who may face barriers to traditional mental health services. The intervention ran between January and March 2025, with online intervention access maintained until August 2025. Where required, Home-Start provided digital devices. Demographic data for the wider Home-Start population were unavailable, as these were not routinely collected.

### 2.3. Participants

#### 2.3.1. Eligibility Criteria

Minimal exclusion criteria were applied to reflect community-level implementation, mirroring the approach taken in the predecessor trial. Eligible participants were Home-Start service users aged 18 or over, living in the UK, who were parents or primary carers of a child aged 2–11, and who had self-reported high levels of anxiety. All genders and family structures (biological, adoptive, step, or foster) were included. Parents (and children) were not excluded based on physical or mental health, neurodevelopmental conditions, or concurrent emotional support. Exclusion criteria were parents aged under the age of 18 or lacked a digital device.

#### 2.3.2. Recruitment

Recruitment was conducted in partnership with Home-Start. Staff and volunteers displayed study materials and supported parent self-referrals. Parents enrolled via a QR code linking to study information, consent, eligibility screening, and baseline (T1) questionnaires. Staff reviewed materials for suitability, and a single training session was delivered to four staff groups covering key knowledge, recruitment scripts, and referral procedures. Initial consultations with Home-Start highlighted potential barriers for parents (anxiety, mistrust, stigma, and childcare constraints). Recruitment strategies were therefore adapted to include volunteer scripts, optional introductory calls with the research team, and researcher attendance at four parent coffee mornings to build trust.

### 2.4. Intervention

The online intervention (PWA) mirrored the evidence-based in-person course (RCC; Cartwright-Hatton et al., 2011). PWA comprised one brief starter module followed by seven core modules (20–30 min each) incorporating psychoeducational animations and videos, case examples, quizzes, reflective tasks, and practical homework. Content included psychoeducation about anxiety, understanding parenting “hotspots”, emotion coaching, play, reinforcement of brave behaviour, boundary setting, and lifestyle strategies (see Supplementary Table S1).

### 2.5. Procedures

#### 2.5.1. Therapist-Support Condition

Most parents in this arm were recruited through Home-Start coffee mornings, with optional introductory calls offered. Over the 8-week intervention, parents could attend up to three therapist contacts: an initial group session (week 1), a mid-course individual check-in, and a final group session (week 8) (Supplementary Table S2). Three options were offered for the 90 min group sessions (one online evening group, two in-person groups on different days). The mid-intervention session was a 30 min Microsoft Teams call (Microsoft Corporation, Redmond, WA, USA). Parents set personal and module-specific goals, which were reviewed at first and final contacts. Home-Start advised on scheduling and venue decisions to maximise accessibility; one venue was moved to a familiar family centre, where crèche provision and transport were funded by Home-Start for some parents. Sessions were delivered by a trainee clinical psychologist (M.C.) who managed participant communication and data collection. Participants and the researcher were therefore not blinded to study arm allocation.

#### 2.5.2. Self-Guided Condition

Participants in the self-guided condition accessed the online PWA modules remotely and independently. Researcher contact was minimal and limited to recruitment coffee mornings and email reminders for post-intervention and follow-up data collection. Participants were asked to complete the intervention within eight weeks.

### 2.6. Objectives

The study aimed (1) to explore differences in engagement with PWA, assessed via intervention activity and post-intervention measures, between the therapist-support and self-guided arms within the constraints of this small, exploratory, non-randomised design; and (2) to explore the feasibility of delivering PWA within the community-based setting (Home-Start), assessed through recruitment, uptake, self-allocation patterns, retention, and acceptability feedback.

### 2.7. Outcome Measures

Data were collected at baseline (T1), post-intervention (T2; week 8), and 6-month follow-up (T3) via Qualtrics survey software (Qualtrics International Inc., Provo, UT, USA). Available online: https://www.qualtrics.com. Responses were anonymised and stored securely on a University of Surrey server. Where parents had multiple children aged 2–11, the child named first alphabetically was selected. A £10 voucher was provided for T3 completion.

#### 2.7.1. Primary Outcome: Acceptability and Engagement

Data on participant engagement with the PWA intervention were collected. This recorded participant activities such as logging in to the intervention and the number of modules started and completed. A module was recorded as accessed if a participant completed at least one task within that module, and as completed if a participant completed ≥80% of that module’s tasks (Supplementary Table S10). Post-intervention satisfaction questionnaires captured acceptability. Open-text responses were analysed using content analysis (Hsieh & Shannon, 2005).

#### 2.7.2. Secondary Outcome Measures: Child and Parent Anxiety

Child and parent measures used in the predecessor trial (Dunn et al., 2024) were used to allow maximum comparability. 

Parent-reported child anxiety was assessed using the Spence Children’s Anxiety Scale—Parent (SCAS-P; Spence, 1997) for children aged five and over (39 items, 4-point scale; 0 = never to 3 = always), and the Spence Preschool Anxiety Scale (SCAS-Preschool; Spence et al., 2001) for children under five (28 items, 5-point scale). Both measures demonstrate strong validity and reliability (Edwards et al., 2010). Internal consistency is high for both the SCAS-P (Cronbach’s α = 0.90; Brown-Jacobsen et al., 2011) and the SCAS-Preschool (α = 0.92; Edwards et al., 2010).

Parent anxiety was assessed using the Screen for Child Anxiety Related Emotional Disorders—Adult version (van Steensel & Bögels, 2014), a 71-item scale scored on a 3-point scale (0 = almost never to 2 = often), with higher scores indicating greater severity. The measure assesses symptoms across seven anxiety disorder subtypes (panic disorder, generalised anxiety disorder, social phobia, separation anxiety disorder, obsessive–compulsive disorder, posttraumatic stress disorder, and specific phobias). The clinical threshold of ≥30 for females (and ≥20 for males) was derived via ROC analysis against ADIS-IV-L diagnoses in a community sample of 327 parents (sensitivity = 0.66, specificity = 0.78 for females, sensitivity = specificity = 0.72 for males; van Steensel & Bögels, 2014). Internal consistency for the SCARED-A total score is high (α = 0.94; van Steensel & Bögels, 2014). The published reliability coefficients and normative standard deviations for each measure (SCARED-A, SD = 16.68; SCAS-Preschool, SD = 19.0; SCAS-P, SD = 16.4) were used to derive the reliable change indices reported in Section 2.8 and Section 3.5 (Supplementary Tables S11–S13).

#### 2.7.3. Tertiary Outcome: Goal Attainment (Therapist-Support Arm)

Parents identified one personal and one module-specific goal in the initial session, rated 0–10 (0 = not met; 10 = fully met). Goal progress was then reviewed in the final session.

#### 2.7.4. Demographic Information

Demographic information was collected for parents (age, gender, ethnicity, financial status, education, mental health and social care history, single-parent status) and their child (age, gender, ethnicity, neurodevelopmental diagnosis, current and past mental health or social care involvement).

#### 2.7.5. Feasibility and Acceptability

Feasibility indicators (recruitment, uptake, self-allocation, retention, and measure completion) were tracked, with reasons for non-participation recorded where available. Acceptability was evaluated through post-intervention feedback forms, engagement metrics, and attrition.

### 2.8. Statistical Analysis

Given the small analytic sample (*n* = 11 with post-intervention data), inferential tests were not appropriate. Analyses focused on descriptive statistics (means, standard deviations, percentage change) for engagement, goal attainment, and anxiety measures across T1–T3 at the group level, complemented by individual-level reliable change analysis for anxiety measures (below).

To examine change at the individual rather than group level, reliable change was calculated for each participant with repeated data using the reliable change index (RCI; Jacobson & Truax, 1991), defined as the pre–post difference divided by the standard error of the difference (SE_diff = √2 x SD x √(1 − *r*)). SD is the normative standard deviation, and *r* is the published internal consistency reliability of each measure (SCARED-A: SD = 16.68, α = 0.94; SCAS-Preschool: SD = 19.0, α = 0.92; SCAS-P: SD = 16.4, α = 0.90). As higher scores indicate greater anxiety, an RCI below −1.96 denotes reliable improvement and above +1.96 reliable deterioration (*p* < 0.05). RCIs were calculated separately for each measure from T1 to T2 and from T1 to T3, and numbers showing reliable improvement, no change, or deterioration are reported in place of a group-mean trajectory.

As all Qualtrics items were mandatory, no missing data procedures were required. Quantitative analyses were conducted in IBM SPSS Statistics (Version 29; IBM Corp., Armonk, NY, USA). A power analysis (G*Power, Version 3.1.9.7 [Computer Software] Heinrich-Heine-Universität Düsseldorf. https://www.psychologie.hhu.de/arbeitsgruppen/allgemeine-psychologie-und-arbeitspsychologie/gpower, accessed on 22 June 2026; Faul et al., 2007) indicated that a future between-group comparison (two-tailed, α = 0.05, 80% power, medium effect size d = 0.5) would require approximately 134 participants (67 per group).

## 3. Results

### 3.1. Participant Flow

**Figure 1:**
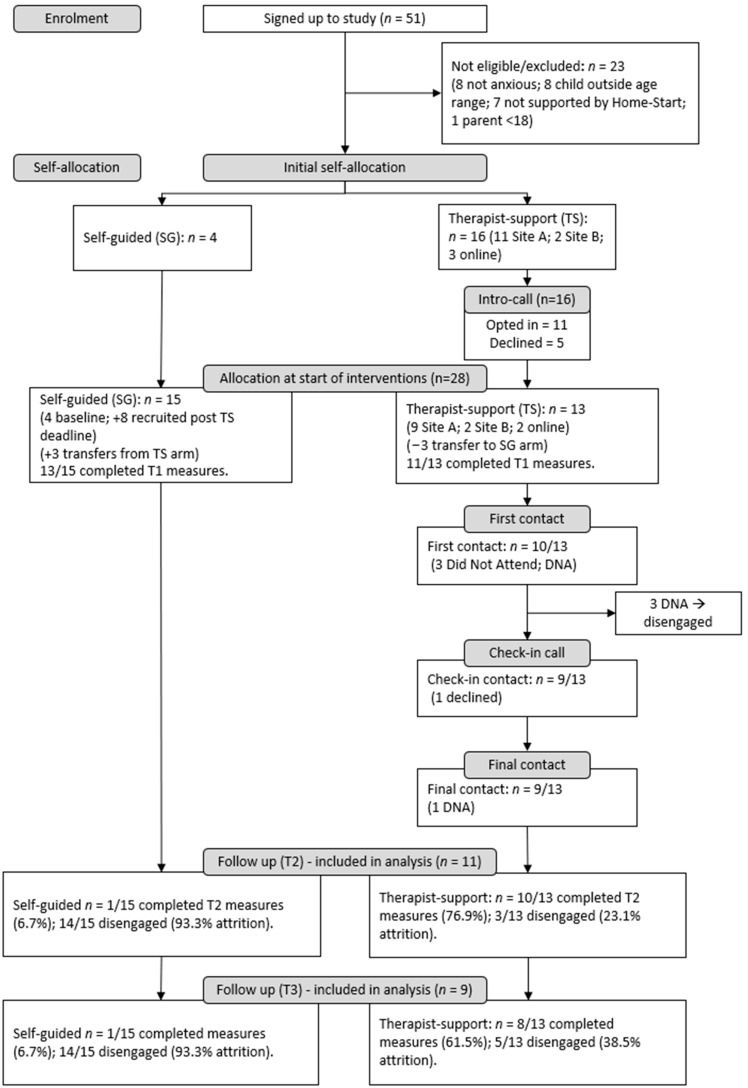
Participant flow diagram. Three therapist-support parents transitioned to self-guided arm. ‘Disengaged’ refers to study withdrawal. Two therapist-support participants who never logged in (P03, P08) still completed T2 outcome measures and are reflected in the 10/13 figure. Supplementary Tables S9 and S10 (Part B) present individual-level engagement and outcome data.

Between January and August 2025, 51 parents expressed interest in the PWA online course. Of these, 28 (55%) consented, met eligibility criteria, and were self-allocated to the therapist-support (*n* = 13) or self-guided arm (*n* = 15), as can be seen in Figure 1 for participant flow.

In the self-guided arm, four parents self-allocated to this option initially; three transferred from the therapist-support arm due to scheduling or childcare constraints; and eight enrolled after the therapist-support recruitment closed in March 2025 (the unguided arm remained open for an additional month).

Attendance varied across the three therapist-support contacts. At the initial session, 3/13 parents did not attend (DNA; 1 = too anxious, 1 = other commitment, 1 = childcare); all three subsequently disengaged from the intervention. At the mid-course check-in, one parent declined to participate as they reported to be managing well with the online modules. At the final therapist-support contact, one parent did not attend due to travel issues. At post-intervention (T2; week 8), 10/13 (76.9%) in the therapist-support arm and 1/15 (6.7%) in the self-guided arm completed outcome measures. At six-month follow-up (T3), completion was 8/13 (61.5%) vs. 1/15 (6.7%). Recruitment was strongest with the researcher attending existing parent groups. The weekly Special Educational Needs and Disabilities (SEND) support group accounted for 14/28 eligible sign-ups. The remainder were recruited via other parent groups (*n* = 2) and self-referrals (*n* = 12), likely from Home-Start volunteer/staff signposting.

### 3.2. Participant Characteristics

#### 3.2.1. Parents

The mean parent age was 35 years (SD = 6.7). Most were female (*n* = 27/28). Thirteen parents (46.4%) identified with a minoritised ethnic background (Mixed, *n* = 2; Asian, *n* = 6; Black/British/African, *n* = 4; Arab, *n* = 1). Half reported financial strain (‘struggling’: 14/28). Educational backgrounds varied (school-leaving age 16: *n* = 3, college: *n* = 12, university: *n* = 13). Anxiety levels were high, as 22/24 parents who completed the anxiety measures scored at or above clinical cut-offs on the SCARED-A (M = 68.0, SD = 30.9; clinical threshold ≥20 points for males and ≥30 points for females; van Steensel & Bögels, 2014). Half (*n* = 14) had received anxiety treatment in the past.

#### 3.2.2. Children

Children ranged from 2 to 10 years of age (M = 2.9; SD = 2.4); most were under 5 years old (23/28). Eighteen were female and 10 were male. Five children had a formal developmental diagnosis and eight were awaiting assessment. Ten had previous involvement with services (e.g., Child and Adolescent Mental Health Services [CAMHS], social care); seven were currently engaged. Many showed elevated anxiety symptoms; on the SCAS-P (SCAS-P; Spence, 1997), 7/12 aged ≥5 scored in the elevated range at T1 (M = 50.7, SD = 15.2), while on the SCAS-Preschool (Spence et al., 2001), 9/16 scored ≥34, indicating elevated anxiety at T1 (M = 33.3, SD = 23.6). 

#### 3.2.3. Demographic Asymmetries Between Arms 

Examination of baseline characteristics by arm (Table 1 and Table 2) suggests some descriptive asymmetries, although the small sample size precludes formal statistical comparison. Financial hardship was broadly similar across arms. Both parents’ reporting ‘comfortable’ financial status were in the self-guided arm; ‘struggling’ was evenly distributed (7 TS vs. 7 SG), and ‘managing’ was slightly more common in the therapist-support arm (6 TS vs. 5 SG). Regarding family structure, two variables were collected: whether the parent lived with the other parent of the index child, or if they self-identified as a single parent (Table 1). Not living with the other parent was comparably distributed across arms (self-guided: 6/15, 40%; therapist-support: 6/13, 46%), while self-identified single-parent status was somewhat more common in the therapist-support arm (5/13, 38%; TS vs. 3/15, 20%; SG). Education was comparatively balanced (university educated: 6/13 vs. 7/15). Among children, current service involvement (5/13 vs. 2/15) and developmental disability status were also more concentrated in the therapist-support arm. This likely reflects delivery context rather than the support condition itself; one group venue was relocated to the SEND parent support group’s regular meeting location in response to access constraints these families had raised, and the resulting familiar venue, staff, and crèche provision plausibly facilitated participation among families with developmental difficulties specifically. This should therefore be read as a feature of the accommodated delivery context (Section 4.3) and not as evidence that therapist-supported delivery itself attracts higher-need families.

### 3.3. Primary Outcome: Acceptability and Engagement

#### 3.3.1. Retention

Retention was substantially higher in the therapist-support arm (Table 3). At T2, 76.9% (10/13) provided outcomes compared to 6.7% (1/15) in the self-guided arm. At T3, retention was 61.5% (8/13) vs. 6.7% (1/15).

#### 3.3.2. Engagement with the Online PWA Course

As arms were not randomly assigned and differed in recruitment pathway and timing (see Section 4.5 ‘Limitations’), the figure below describes two non-equivalent groups; therapist-supported delivery was associated with higher engagement in this implementation context, but this cannot be interpreted as a demonstrated effect of therapist support. 

**Figure 2:**
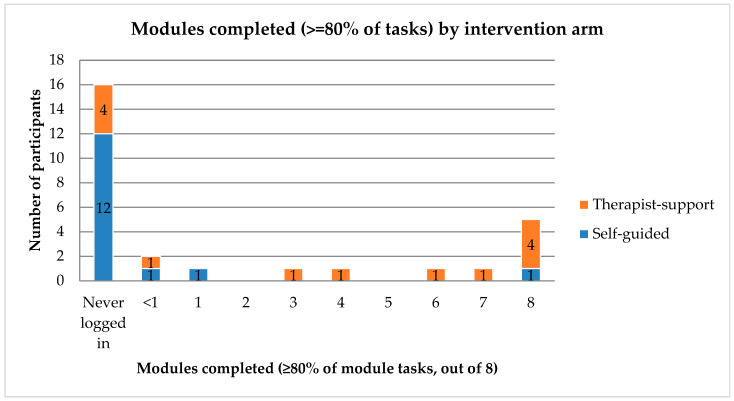
Distribution of modules completed (≥80% of module tasks, out of eight) by intervention arm. Bars show the number of participants completing each number of modules, stacked by arm. “Never logged in” indicates no platform access; “<1” indicates participants who accessed the starter module but completed less than 10% of its tasks before disengaging.

Engagement differed markedly by arm and followed a bimodal pattern (Figure 2), where most participants either did not engage with the platform at all or completed the course in full, with very few falling in between. All figures refer to the primary completed modules measure (≥80% of module tasks; see Section 2.7.1); the accessed measure (≥1 task) gave near-identical results and is reported alongside in Supplementary Table S10. 

Overall, 16/28 participants (57%) never logged in. A further 2/28 (7%) accessed the platform but completed fewer than 10% of the starter module tasks and are represented as <1 in Figure 2. At the other end, 5/28 (18%) completed all eight modules, and the remaining 5/28 (18%) showed partial completion across 1–7 modules. 

In the self-guided arm (*n* = 15), 12 (80%) never logged in; one accessed the starter module only (<1); one completed a single module; and one completed all eight. In the therapist-support arm (*n* = 13): four (31%) never logged in; one accessed the starter module only (<1); four showed partial completion (3, 4, 6 and 7 modules respectively); and four completed all eight modules. 

Given the bimodal pattern and small sample size, means should not be read as a description of a typical participant in either arm. For descriptive comparability with the predecessor trial, the mean number of modules completed (≥80%) was 2.2 (SD = 3.3) overall, 4.0 (SD = 3.6) in the therapist-support arm, and 0.6 (SD = 2.1) in the self-guided arm.

### 3.4. Acceptability (Satisfaction)

Eight out of 13 therapist-supported parents returned feedback forms. Seven were ‘very happy’ with the course and seven strongly agreed they would recommend it. All reported high satisfaction with therapist support. Qualitative feedback reflected five main themes (Supplementary Tables S3 and S4): peer connection/normalisation (*n* = 7/8); therapist encouragement/accountability (*n* = 6/8); positive affect, e.g., improved self-esteem and validation (*n* = 4/8); practical skill development (*n* = 4/8); and accessible content (*n* = 4/8). Reported access enablers included flexible delivery, mobile access, and ‘bite-size’ modules. Some barriers were reported related to childcare, technical issues, and competing demands. No feedback was received from the self-guided arm.

### 3.5. Secondary Outcomes: Anxiety

#### 3.5.1. Child Anxiety

Patterns differed by measure and age group; given the small sample sizes, results are treated cautiously and reported separately by measure. Across both child measures, no consistent direction of change was observed.

*SCAS-Preschool (<5 years; n = 7 at T2–T3).* Mean scores remained stable from T1 to T2 (M = 44.43) and slightly decreased by T3 (M = 39.57, SD = 12.62). Reliable change analysis (Table S12) showed no child reliably improving from T1 to T2 and one reliable deterioration (1/7); by T3, 3/7 children showed reliable improvement, 2/7 showed reliable deterioration, and two showed no reliable change.

*SCAS-P (≥5 years; n = 4 at T2, n = 2 at T3).* Mean scores rose from T1 (M = 59.50, SD = 7.55; *n* = 4) to T2 (M = 65.50, SD = 18.30; *n* = 4) before falling at T3, where only two children were assessed (*n* = 2); their scores (62 and 43) are reported individually rather than as a mean. Reliable change analysis (Table S13) showed one child with reliable deterioration from T1 to T2 (1/4), and neither of the two children with follow-up data showed reliable change by T3.

#### 3.5.2. Parent Anxiety

SCARED-A (parental anxiety; *n* = 11 at T2, *n* = 9 at T3). Mean scores decreased from 73.09 (SD = 17.14) at baseline (T1) to 70.18 (SD = 28.82) at post-intervention (T2; week 8) and to 63.22 (SD = 27.17) at six-month follow-up (T3; Table 4). Given the small, changing denominator, individual reliable change is more informative than the group-mean trajectory. Reliable change analysis (Table S11) showed that from T1 to T2, four of the 11 parents showed reliable improvement, five showed no reliable change, and two showed reliable deterioration (1/11 of whom never accessed the online course, so the increase cannot be attributed to the intervention; the other is interpreted in clinical context in Section 3.8). By T3, four of the nine parents with follow-up data showed reliable improvement, five showed no reliable change, and none showed reliable deterioration (*n* = 9).

### 3.6. Goal Attainment (Therapist-Support; Tertiary Outcome)

Seven parents provided pre- and post-goal ratings (Supplementary Tables S6 and S7). Personal goals were coded as parent wellbeing/self-care (*n* = 4), parent–child interaction/relationship (*n* = 1), and child wellbeing/supporting child anxiety (*n* = 2). Five parents reported progress on personal goals (Δ range = +1 to +7) and two remained unchanged, giving a mean change of +2.9 (SD = 2.7) across all seven. All seven progressed on module-specific goals (Δ range = +3 to +8; M = +5.3, SD = 1.8). 

### 3.7. Completers vs. Non-Completers (Attrition Patterns)

Descriptive comparisons (Supplementary Table S8) indicated that completers (defined as completing T2 measures; *n* = 11) compared with non-completers were more likely to self-identify as single parents (45% vs. 18%; the proportion not living with the other parent was comparable: 45% vs. 41%), have a child with a developmental disability (73% vs. 29%), and have past or current involvement with child services (past: 55% vs. 24%; current: 45% vs. 12%). However, 10 of 11 completers were in the therapist-support arm and the SEND parent support group—which accounted for the largest source of referrals (14/28) and highest attendance at therapist-support contacts following venue relocation—likely explaining the concentration of developmental disability among completers. These patterns reflect recruitment pathway and delivery-context accommodations (Section 3.2.3) rather than independent predictors of completion.

### 3.8. Parent Anxiety Outcomes and Goal-Based Behavioural Change (Therapist-Support Arm)

The reliable change findings reported in Section 3.5.2 are notable given the severity of this sample, as 22 of 24 parents who completed anxiety measures scored at or above clinical cut-offs (M = 68.0, SD = 30.9; ≥20 for males, ≥30 for females; van Steensel & Bögels, 2014), and all 11 with repeated measures exceeded the threshold at baseline (range 51–107). At six-month follow-up, eight of the nine assessed remained above threshold—the exception being the parent with the largest reliable improvement—so although several improved reliably, anxiety remained clinically significant for most. Read alongside progress on module-specific goals (M = +5.3, SD = 1.8; Supplementary Table S7) and personal goals (M = +2.9, SD = 2.7; Supplementary Table S6), this points to a coherent pattern of meaningful engagement in a high-need group. These findings remain exploratory and should not be interpreted as evidence of effectiveness given the small sample.

## 4. Discussion

This exploratory feasibility study examined (1) differences in engagement with the PWA online intervention between therapist-support and self-guided delivery for anxious parents and (2) the feasibility of delivering PWA within a community-based service (Home-Start). As a small, non-randomised feasibility study, the findings reported below are intended to generate hypotheses and inform future research design rather than to establish the effectiveness of either delivery model.

### 4.1. Acceptability and Engagement

Engagement was higher in the therapist-support arm, with participants logging in more frequently, completing more modules, and sustaining participation. As discussed in Section 4.5, this difference cannot be separated from variation in recruitment pathway and baseline motivation between arms; the mechanisms discussed below (supportive accountability, goal-setting, therapeutic alliance) are therefore offered as hypothesised rather than established explanations for the engagement gap. Engagement in the self-guided arm was minimal; most never logged in (80%; 12/15), two accessed a single module, and only one completed all modules. This pattern aligns with the broader DMHI literature showing high attrition without human support (Fleming et al., 2018; Baumel et al., 2019; Wasil et al., 2020). Consistent with the supportive accountability model (Mohr et al., 2011), therapist involvement appeared to foster motivation, accountability, and persistence. A recent meta-analysis of 117 DMHI trials similarly found that guided delivery and a stronger therapeutic relationship were both significantly associated with greater engagement, with the latter effect amplified by longer treatment duration (Zainal et al., 2025), broadly consistent with the pattern observed here across the eight-week course.

Collaborative goal setting and module prioritisation may have strengthened autonomy and relevance (Yardley et al., 2016). All therapist-support participants who set goals demonstrated progress on their module-specific goal, reflecting findings from meta-reviews showing improved engagement and outcomes for human-supported DMHIs (Werntz et al., 2023). Qualitative feedback highlighted relational factors, such as therapist encouragement, validation, and group support, as key to sustaining participation, aligning with evidence that therapeutic alliance supports adherence in digital interventions (Schmidt et al., 2014; Berger, 2017; Metcalfe et al., 2021; Mohr et al., 2011). The small sample precludes any dose–response analysis. At the individual level, parental anxiety improved reliably in a subset (four of 11 at T2; four of nine at T3), while others showed no reliable change; these descriptive findings cannot be attributed to module completion or therapist support given the sample size and confounds (Section 4.5). Some therapist-support parents described increased confidence and calmer parenting at final contacts, though these accounts are anecdotal and limited to engaged parents. Where child anxiety reliably increased (i.e., deteriorated), two non-exclusive explanations are plausible: a short-term, exposure-related rebound as parents reduced accommodation of avoidance, or ongoing environmental adversity (e.g., housing insecurity, financial strain) beyond the reach of a brief parenting intervention.

### 4.2. Comparing with the Predecessor Trial

The present feasibility study intentionally partnered with Home-Start to reach a more diverse group of parents than the predecessor trial, and the resulting sample was indeed more socioeconomically and ethnically diverse (46% from minoritised ethnic backgrounds; 50% experiencing financial hardship; 43% not living with the other parent of the index child; 29% self-identifying as single parents), suggesting that community-embedded delivery and therapy support may broaden reach beyond unguided digital dissemination alone. However, this diversity reflects the community-embedded implementation—including the choice of partner organisation, recruitment pathways (notably the SEND parent group, which accounted for half of eligible sign-ups) and venue with a crèche provision—rather than the therapist-supported model specifically. Since the recruitment pathway and intervention arms were not independently varied, we cannot determine how much of this diversity would be replicated by the therapist-support model alone in a different context. This remains a question for future research. Engagement was also higher among parents who received therapist support, though self-selection into arms means this cannot be interpreted as a demonstrated effect of therapist support independent of recruitment pathway and motivation.

### 4.3. Community-Based Feasibility and Implementation

Delivering PWA within Home-Start was feasible but required collaborative adaptation. Key facilitators included provision of an onsite crèche, accessible venues familiar to parents, and transport support for isolated or highly anxious parents. Flexible delivery (in-person, phone or video), video recording catch-up materials, reminder tools, and ability to reschedule check-in calls appeared to support retention among families facing multiple stressors, although this cannot be tested formally given the sample size. While DMHIs offer scalability and cost efficiency (Kazdin, 2019; Hollis et al., 2017), their effectiveness depends on user engagement. Therapist support increased resource demand; participation from underrepresented groups was observed alongside this delivery model, though as discussed in Section 3.2.3 and Section 4.2, this is more plausibly attributable to specific recruitment and venue accommodations (e.g., the SEND group venue relocation) than to therapist support itself. This observation is nonetheless broadly consistent with research suggesting socially disadvantaged families may benefit from relational and practical support (Katz et al., 2001; Peters et al., 2005). Provision of free childcare—used in other community programmes, such as Empowering Parents, Empowering Communities (Day et al., 2012)—appeared important for attendance in this sample and may warrant consideration in future parent-focused prevention interventions, particularly for single parents and/or parents with preschool-aged children.

### 4.4. Digital Inequalities and Policy Context

Several parents encountered digital barriers (login issues, password resets, and difficulty relocating emailed course link), which were often resolved during therapist contacts, though we cannot establish these prevented withdrawals that would have otherwise occurred. This is consistent with the view that digital exclusion is shaped not only by access but by skills, confidence, and support (Greenhalgh et al., 2017), with NHS England’s Inclusive Digital Healthcare Framework (NHS England, 2023), whose four pillars—access, skills, motivation, and trust—were all reflected in the difficulties parents reported. The absence of feedback from the self-guided arm limits understanding of their disengagement; technical barriers are one plausible but unconfirmed contributor. 

At a policy-level, the NHS’s long-term plan—Fit for the Future (Department of Health and Social Care et al., 2025)—promotes digital innovation while cautioning that, without inclusive design, digital tools may exacerbate inequalities (McAuley, 2014; Farre et al., 2023), echoing the ‘digital health divide’ literature, which distinguishes inequalities in access, skills, and outcomes and highlights community-embedded delivery as a strategy for narrowing these gaps (Western et al., 2025). The present observations speak only tentatively to this, as PWA’s mobile-friendly, “bite-sized” format was described as helpful by some engaged completers (though not generalisable to the wider sample), while minimal self-guided engagement may illustrate how self-directed delivery can disadvantage families facing multiple barriers—albeit inseparable from that arm’s composition and recruitment pathway (Section 4.5). Taken together, and requiring confirmation in a design capable of establishing causal effects, this points to the potential of blended models combining scalable digital content with minimal, strategically deployed human support and trusted community partnerships (Western et al., 2025; Hollis et al., 2017).

### 4.5. Limitations and Generalisability

Interpretation is limited by the small non-randomised and non-blinded sample. A key limitation is participant self-allocation into arms rather than by randomisation, compounded by differential recruitment; most therapist-support parents were recruited face-to-face via Home-Start coffee mornings, while the self-guided arm partly comprising parents who transferred out due to scheduling or childcare constraints (*n* = 3) or enrolled later via more passive signposting after recruitment closed (*n* = 8; Section 3.1). 

The engagement gap—most self-guided parents never logging in (80%; 12/15) versus a markedly higher proportion of completed modules in the therapist-supported arm—may reflect therapist contact, baseline motivation, recruitment pathway, or their combination; as no baseline motivation or digital literacy measure were collected prior to allocation, these cannot be disentangled with the present data. Self-allocation was, however, a deliberate design choice for this feasibility study, as it reflects how families would access support in real-world implementation, whereas imposing randomisation on a vulnerable, help-seeking community sample risked undermining engagement and acceptability—the very outcomes under investigation. Additionally, Home-Start’s established trusting relationships with many families likely aided engagement but may be hard to reproduce in settings lacking comparable relational infrastructure.

Attrition was substantial in the self-guided arm, limiting comparative analysis with feedback only obtained from engaged participants. Because the same researcher (M.C.) delivered the therapist-support sessions, managed participant communication, and collected outcome and satisfaction data without blinding, there is meaningful potential for demand characteristics and courtesy bias, particularly in the satisfaction ratings and the self-reported goal-attainment scores (0–10), which were collected directly by the clinician who provided the support. These findings should therefore be interpreted with appropriate caution and as a feature of this specific implementation context. Notably, S.C.-H., the developer of the PWA intervention, was not involved in the study design, methodology, data collection, or analysis, and reviewed the written manuscript only after the intervention and data collection had been completed, limiting the potential for this conflict of interest to have influenced the conduct of the study itself.

Nevertheless, real-world implementation embedded within a community service enhances ecological validity and provides valuable insight into engagement among families with higher levels of social need. Generalisability beyond this setting remains uncertain and replication across other community contexts is required. No adverse effects were identified, though technical challenges initially caused frustration for some parents.

## 5. Conclusions

This feasibility study suggests that organisational accommodations (a familiar venue, creche and transport support) helped reduce barriers to participation for these parents to access the intervention, while therapist support coincided with sustained engagement once involved. Although the relative contribution of each cannot be established with this design, the most striking observation is that 80% of the self-guided arm never logged in, suggesting that delivery without support in this context, as in the predecessor trial, would have reached only a minimal proportion of these parents. This small study shows that a community-embedded, therapist-supported model can enable parents typically underrepresented in digital intervention research to access and complete the course. 

Future research should test these accommodation and support components separately, ideally through randomisation or matched recruitment routes, and be prospectively designed around an implementation science framework (e.g., RE-AIM or CFIR) to enable systematic analysis of the conditions and mechanisms underlying feasibility.

## Figures and Tables

**Table 1:** Baseline parent demographics by intervention arm and overall sample.

Parent Demographics	Self-Guided	Therapist-Support	Overall
Age, mean (SD)	35 (6.7)	35 (6.9)	35 (6.7)
Gender			
Female	14	13	27
Male	1	0	1
Ethnicity			
White	8	7	15
Mixed	0	2	2
Asian	4	2	6
Black	2	2	4
Arab	1	0	1
Financial status			
Comfortable	2	0	2
Managing	6	5	11
Struggling	7	7	14
Treatment for anxiety in past 12 months			
Yes	7	7	14
No	8	6	14
Education			
Left school at 16	2	1	3
Completed college	6	6	12
Completed university	7	6	13
Lives with other parent of index child			
Yes	9	7	16
No	6	6	12
Identifies as single parent	3	5	8

**Table 2:** Baseline child demographics by intervention arm and overall sample.

Child Demographics	Self-Guided	Therapist-Support	Overall
Age, mean (SD)	2.6 (2.1)	3.3 (2.7)	2.9 (2.4)
Gender			
Female	9	9	18
Male	6	4	10
Ethnicity			
White	7	8	15
Gypsy/traveller	0	1	1
Mixed	1	1	2
Asian	4	2	6
Black	2	1	3
Arab	1	0	1
Treatment for anxiety in past 12 months			
Yes	2	0	2
No	13	15	26
Developmental disability			
Yes—formal diagnosis	1	4	5
Yes—awaiting assessment	4	4	8
No	7	5	12
Not sure	3	0	3
Previous involvement with other services			
Yes	5	5	10
No	10	8	18
Current involvement with other services			
Yes	2	5	7
No	13	8	21

**Table 3:** Retention by time point and arm.

Time Point	Overall *n* (%)	Therapist-Support *n* (%)	Self-Guided *n* (%)
T2 (8 weeks post-intervention start)	11/28 (39.2)	10/13 (76.9)	1/15 (6.7)
T3 (~6 months after T2)	9/28 (32.1)	8/13 (61.5)	1/15 (6.7)

Note: denominators are all enrolled participants in each arm (therapist-support *n* = 13, self-guided *n* = 15). Retention reflects completion of any anxiety outcome measure at the time point.

**Table 4:** Means and standard deviations for anxiety measures (T1–T3)—repeated measures sample.

Measure	Time Point	*n*	Mean	SD
SCARED-A Total (parental anxiety)	T1	11	73.09	17.14
	T2	11	70.18	28.82
	T3	9	63.22	27.17
SCAS-Preschool (child anxiety; <5 years)	T1	7	44.43	25.47
	T2	7	44.43	23.06
	T3	7	39.57	12.62
SCAS-P (child anxiety; ≥5 years)	T1	4	59.50	7.55
	T2	4	65.50	18.30
	T3	2	Scores: 62 and 43	-

## Data Availability

The original contributions presented in this study are included in the article/Supplementary Materials. The raw individual-level data are not publicly available, as participants did not provide consent for public data sharing; disclosure would compromise participant confidentiality and the terms of the informed consent obtained. Further inquiries can be directed to the corresponding author.

## Supplementary Materials

The following supporting information can be downloaded at: https://www.mdpi.com/article/10.3390/bs16071149/s1. Table S1: Module overview of the Raising Confident Children (PWA) intervention; Table S2: Overview of therapist-support arm contacts, showing the three format options offered for the initial and final sessions to enhance accessibility following consultation with Home-Start; Table S3: Conventional content analysis of open-text responses from feedback forms. Table shows themes, theme definitions and example quotes; Table S4: Coded responses using conventional content analysis; Table S5: Comparison of anxiety outcomes between current feasibility study and Dunn et al. (2024) Randomised Controlled Trial; Table S6: Therapist-support arm: individual personal goals. Group mean and standard deviation reported; Table S7: Therapist-support arm: individual module-specific goals. Group mean and standard deviation reported; Table S8: Parent and child demographics by completion status (completers *n* = 11; non-completers *n* = 17); Table S9: Individual-level SCARED-A (parent anxiety) raw scores and change scores across time points (T1 = pre-treatment, T2 = post-treatment, T3 = follow-up). Engagement with the online course was operationalised as the number of modules completed (0–8); Table S10: Engagement measures: operational definitions, rationale and comparison of “accessed” vs. “completed” outcomes by intervention arm; Table S11: Individual SCARED-A (parent anxiety) scores and reliable change index (*n* = 11); Table S12: Individual SCAS-Preschool (child anxiety, <5 years) scores and reliable change index (*n* = 7); Table S13: Individual SCAS-P (child anxiety, ≥5 years) scores and reliable change index (T2; *n* = 4; T3 *n* = 2).

## Abbreviations

The following abbreviations are used in this manuscript:

CAMHS	Child and Adolescent Mental Health Services
CBT	Cognitive Behavioural Therapy
DMHI	Digital Mental Health Intervention
DNA	Did Not Attend
PWA	Parenting with Anxiety (Online Intervention)
RCC	Raising Confident Children (Face-To-Face Programme)
RCT	Randomised Controlled Trial
SCARED-A	Screen for Child Anxiety Related Emotional Disorders—Adult Version
SCAS-P	Spence Children’s Anxiety Scale—Parent Report
SCAS-Preschool	Spence Preschool Anxiety Scale
SEND	Special Educational Needs and Disabilities
