# Supplementary material for: Exploring Engagement with the Online ‘Parenting with Anxiety’ Intervention Within a Community Setting: A Quasi-Experimental Feasibility Study
Source: Behav Sci (Basel). 2026 Jul 8;16(7):1149. doi: 10.3390/bs16071149 (PMC13405733; doi:10.3390/bs16071149)
Supplement: Supplementary file 1 [file behavsci-16-01149-s001.zip › behavsci-4350273-supplementary.pdf]

SUPPLEMENTARY TABLES

Exploring Engagement with the Online 'Parenting with Anxiety' Intervention within a Community Setting: A Quasi-Experimental Feasibility Study

Carter, M.; Frith, H.; Cartwright-Hatton, S; Dunn, A.

Table S1. Module overview of the Raising Confident Children (PWA) intervention.

**Module title**	**Summary**
Starter Module	All about anxiety and confidence in children
Module 1: The Comfort Zone	How avoidance maintains anxiety and how to reduce it
Module 2: Where are your hot spots?	Understanding parent hotspots: overprotection and perfectionism
Module 3: Good Stress, Bad Stress and Overprotection	Modelling confident behaviour, compensating for anxiety-driven gaps
Module 4: Emotion Coaching	Tuning into child's emotions to help with regulation and attunement
Module 5: The Playful Parent	The importance of child-directed play in building confidence and relationships
Module 6: Get More Good and Brave Behaviour	Skills and strategies to reinforce helpful and brave behaviours
Module 7: Boundary Building — Setting Clear Limits for Children	Positive behaviour strategies to help with discipline and boundaries
Module 8: Mind and Body	Psychoeducation on sleep, exercise and diet

Table S2. Overview of therapist-support arm contacts, showing the three format options offered for the initial and final sessions to enhance accessibility following consultation with Home-Start.

**Therapy contact**	**Overview**
Initial Session (week 1)<br><br>Option 1: Online, evening<br>Option 2: In-person, Guildford<br>Option 3: In-person, Elmbridge	Introduced the course, reviewed the starter module, and delivered psychoeducation using group tasks to normalise experiences. A simple CBT formulation (thoughts–feelings–behaviours) was linked to the seven confident thoughts underpinning the RCC programme. Parents identified a priority module and set both a personal and module-specific goal (rated pre/post). Barriers to engagement were explored and problem-solved.
Mid-way check-in (Week 3–4)	A 30-minute virtual one-to-one call reviewed goals, offered encouragement, and tailored strategies to family needs.
Final session (week 8)<br><br>Option 1: Online, evening<br>Option 2: In-person, Guildford<br>Option 3: In-person, Elmbridge	Parents reflected on progress, revisited their goals, and discussed strategies for maintaining change, supported by a reflective exercise on personal growth.

Table S3. Conventional content analysis of open-text responses from feedback forms. Table shows themes, theme definitions and example quotes.

Theme	Definition	Example quotes
Peer normalisation & group connection	Feeling less alone; value of sharing/hearing other parents; welcoming group climate.	"The groups were powerful and it was lovely to share my story and hear other people who have gone through the same life experiences."<br><br>"I loved the idea of the group sessions and felt very welcomed."<br><br>"The course has helped me realise that I am not a bad parent. I feel less alone now that I have done the course. It would not have been the same without the groups, as hearing other people's stories has helped and having the emotional support."<br><br>"The groups were really helpful and I liked meeting other parents going through similar hardships."
Therapist support & gentle accountability	Check-ins/contacts helped focus, stay on track; felt supported; problem-solving.	"Really liked the check-in call, as I was struggling to find the time to do the modules but [therapist] helped me work out what to focus on and get back on track."<br><br>"I don't think I would have been able to complete the course if there was not someone to help me through it."<br><br>"Having a human person makes the course more real and easier to engage with. I would not have engaged without this as there is no accountability. I found having the psychologist available during the course lessened my anxiety."<br><br>"[therapist] was very kind and helped me problem-solve my difficulties. She made me feel less alone and that how I was feeling mattered."
Positive emotional impact/self-compassion	More hope/confidence; being kinder to self; feeling listened to.	"I have more hope that I can be a better parent."<br><br>"It taught me to be kind to myself."

Practical strategies & increased knowledge	Concrete tools/skills; 'doable' steps; using modules/ideas with child; increased knowledge of anxiety.	"This course was an eye opener. It helped me understand that there are strategies to help understand my child's behaviour."<br><br>"I have also used some of the skills and seen how they work which has helped me feel more confident."<br><br>"I have really enjoyed the course and have learnt so much. The overprotection module and hotspots were really interesting and have helped me approach parenting differently. I have noticed when I let go more, my child feels more confident."
Access enablers	Childcare/transport, phone access, flexible format.	"I appreciated the phone call, face-to-face and then online. I am a single parent so without that I would not have been able to do the course."<br><br>"It is accessible. I don't have a laptop either so I could do everything on my phone and fit it within my day."
Access barriers	Childcare; initial technical difficulties.	"If there was childcare offered in [location] for face-to-face options."<br><br>"Test the website first before sending it to parents as if I did not have help then I would have dropped out."
Format & tech usability	"Bite-size" course, helpful videos/representation; login issues and fixes.	"The course was bitesize, like GCSE bitesize, and it was easy to digest. The videos were really useful."
Suggestions for improvement	More child-facing stories; extra follow-up/group; test website first; preference for individual in-person sessions.	"It might be good to have another follow-up session to see how we are getting on."<br><br>"I liked the idea of having more stories to share with your children like the volcano story and maybe another group session to help more parents."

Goal setting/progress	Valued setting a goal; pride/progress on personal/module goals.	"I liked setting a goal as I felt really proud of myself for being able to do it."<br><br>"I feel proud of completing the course and working towards my goal with my daughter. I have been out for the first time in ages which makes me happy."

Table S4. Coded responses using conventional content analysis.

ID	Peer normalisation & group connection	Therapist support & accountability	Format & tech usability	Positive emotional impact	Practical strategies	Access enablers	Access barriers
P1	✓	✓		✓	✓		✓ (tech issues)
P2	✓	✓	✓	✓	✓	✓ (flexible approach)	
P3	✓	✓	✓				✓ (childcare required)
P4	✓						
P5	✓			✓	✓		
P6	✓	✓	✓			✓ (access on phone)	
P7	✓	✓	✓	✓	✓	✓ (module format)	✓ (tech issues)
P8		✓	✓			✓ (flexible approach)	✓ (other commitments)
Total	7	6	5	4	4	4	4

Table S5. Comparison of anxiety outcomes between current feasibility study and Dunn et al. (2024) Randomised Controlled Trial.

Measure	Time point	RCC Feasibility Study (Home-Start) Mean (SD)	Dunn et al. RCT (2024) Effect size (Cohen's d)	Direction of change
Parent anxiety (SCARED-A)	*T1 (Baseline)*	*73.09 (17.14)*	—	*High baseline anxiety in both samples*
	T2 (Post-intervention; Week 8)	*70.18 (28.82)*	*$d = -0.17$, $p < .001$*	*↓ Slight reduction*
	T3 (6-month follow-up)	*63.22 (27.17)*	*$d = -0.14$ ($p < .001$), maintained to 9–25 months*	*↓ Sustained improvement*
Child anxiety (SCAS-Preschool; <5 years)	*T1 (Baseline)*	*44.43 (25.47)*	—	—
	T2 (Post-intervention; Week 8)	*44.43 (23.06)*	*$d = -0.16$, $p < .001$*	*↓ Modest reduction/sustained*
	T3 (6-month follow-up)	*39.57 (12.62)*	*$d = -0.15$ ($p < .001$), maintained*	*↓ Sustained lower anxiety*
Child anxiety (SCAS-P; ≥5 years)	*T1 (Baseline)*	*59.50 (7.55)*	—	—
	T2 (Post-intervention; Week 8)	*65.50 (18.30)*	*$d = -0.16$, $p < .001$*	*↑ Slight increase (n=4)*
	T3 (6-month follow-up)	*Scores: 62, 43 (n=2; mean not reported)*	*$d = -0.15$ ($p < .001$), maintained*	*↓ Reduction (n=2 only)*

** Feasibility T2 = 8 weeks; T3 = 6-month follow-up. Dunn et al. (2024) T2 = 6 months; T3 = 9–25 months. In the feasibility study, SCAS-P and SCAS-Preschool scores were analysed separately due to small subgroup sizes; SCAS-P T3 n=2 so individual scores are reported rather than a mean. In Dunn et al. (2024), these were standardised and combined for analysis across the full child age range (2–11 years). Cohen's d values: approximately 0.2, 0.5, and 0.8 are conventionally interpreted as small, medium, and large effects, respectively.*

Table S6. Therapist-support arm: individual personal goals. Group mean and standard deviation reported.

Participant ID	Goal category	Pre	Post	Goal difference
P07	Parent wellbeing/self-care	3	10	+7
P05	Parent wellbeing/self-care	1	6	+5
P06	Parent–child interaction/relationship	3	7	+4
P09	Child wellbeing/supporting child anxiety	3	6	+3
P10	Child wellbeing/supporting child anxiety	2	3	+1
P01	Parent wellbeing/self-care	3	3	0
P02	Parent wellbeing/self-care	4	4	0
**Mean (SD)**				**+2.9 (2.7)**

Table S7. Therapist-support arm: individual module-specific goals. Group mean and standard deviation reported.

Participant ID	Module	Pre	Post	Goal difference
P06	What are my hotspots?	0	8	+8
P01	Boundary Building	2	9	+7
P05	Boundary Building	2	8	+6
P02	Playful Parent	1	6	+5
P07	What are my hotspots?	3	7	+4
P09	Playful Parent	3	7	+4
P10	What are my hotspots?	2	5	+3
**Mean (SD)**				**+5.3 (1.8)**

Table S8. Parent and child demographics by completion status (completers n = 11; non-completers n = 17). The table summarises a missingness analysis comparing families who engaged with the online intervention and completed measures with those who dropped out.

Variable	Completers (n = 11)	Non-completers (n = 17)	Total (N = 28)
Parent age, Mean (SD)	34.8 (8.0)	36.1 (5.8)	35 (6.7)
Gender			
Female	11 (100%)	16 (94%)	27 (96%)
Male	0 (0%)	1 (6%)	1 (4%)
Ethnicity			
White	6 (55%)	9 (53%)	15 (54%)
Minoritised	5 (45%)	8 (47%)	13 (46%)
Financial status			
Comfortable/Managing	5 (45%)	8 (47%)	13 (46%)
Struggling	6 (55%)	9 (53%)	14 (54%)
Anxiety treatment past 12 months			
Yes	5 (45%)	9 (53%)	14 (50%)
No	6 (55%)	8 (47%)	14 (50%)
Education			
≤College	7 (64%)	8 (47%)	15 (54%)
University	4 (36%)	9 (53%)	13 (46%)
Live with other parent			
Yes	6 (55%)	10 (59%)	16 (57%)
No	5 (45%)	7 (41%)	12 (43%)
Single parent			
Yes	5 (45%)	3 (18%)	8 (29%)
No	6 (55%)	14 (82%)	20 (71%)
Child age, Mean (SD)	3.7 (2.7)	2.4 (2.0)	2.9 (2.4)
Child ethnicity			
White	7 (64%)	8 (47%)	15 (54%)
Minoritised	4 (36%)	9 (53%)	13 (46%)
Child developmental disability			
Yes (formal/suspected)	8 (73%)	5 (29%)	13 (46%)
No	3 (27%)	12 (71%)	15 (54%)
Child anxiety treatment past 12 months			

Yes	0 (0%)	2 (12%)	2 (7%)
No	11 (100%)	15 (88%)	26 (93%)
Past involvement with other services			
Yes	6 (55%)	4 (24%)	10 (36%)
No	5 (45%)	13 (76%)	18 (64%)
Current involvement with other services			
Yes	5 (45%)	2 (12%)	7 (25%)
No	6 (55%)	15 (88%)	21 (75%)

Ethnicity collapsed to White vs. Minoritised; financial status collapsed to Comfortable/Managing vs. Struggling; education collapsed to University vs. ≤College; child developmental disability collapsed to Yes (formal or suspected) vs. None/Not sure. Completer defined as the 11 participants who completed T2 measures.

Table S9. Individual-level SCARED-A (parent anxiety) raw scores and change scores across time points (T1 = pre-treatment, T2 = post-treatment, T3 = follow-up). Engagement with the online course was operationalised as the number of modules completed (0–8).

PID	Arm	Engagement status	T1	T2	T3	ΔT2–T1	ΔT3–T1
P01	Therapist-support	Engaged partially (4/8)	107	87	86	-20	-21
P02	Therapist-support	Engaged fully (7/8)	56	41	47	-15	-9
P03	Therapist-support	No engagement/did not log in	66	94	—	+28	
P04	Therapist-support	Engaged partially (3/8)	57	68	—	+11	
P05	Therapist-support	Engaged fully (8/8)	94	100	86	+6	-8
P06	Therapist-support	Engaged fully (8/8)	81	71	65	-10	-16
P07 *	Therapist-support	Engaged fully (8/8)	78	106	88	+28	+10
P08	Therapist-support	No engagement/did not log in	70	60	73	-10	+3
P09	Therapist-support	Engaged mostly (6/8)	63	49	40	-14	-23
P10	Therapist-support	Engaged fully (8/8)	51	10	7	-41	-44
P11	Self-guided	Engaged fully (8/8)	81	86	77	+5	-4

*ΔT2–T1 and ΔT3–T2 represent change from pre- to post-treatment and post-treatment to follow-up respectively; negative values indicate symptom improvement. Missing values are denoted by "—". Given the very small sample (N = 11) and uneven group sizes (10 in therapist-support vs. 1 in self-guided), these results are descriptive only and should not be interpreted as evidence of effectiveness. * P07 showed an increase in anxiety scores post-intervention; however, this should be interpreted in context. At baseline, P07 presented with high anxiety and significant agoraphobic difficulties, requiring volunteer support to attend sessions. Despite the elevated score, P07 achieved progress in both personal and module-specific goals and demonstrated increased confidence and functional engagement. The rise in anxiety is understood as reflecting engagement with previously avoided situations (i.e., exposure), rather than deterioration.*

Table S10: Engagement measures: operational definitions, rationale and comparison of "accessed" vs "completed" outcomes by intervention arm.

Part A: Operational definitions

Construct	Definition	Rationale	Precedent
**Platform uptake**	Participant logged in at least once and accessed the starter module	Captures whether the intervention was initiated at all (Zainal et al., 2025)	Equivalent to 'uptake' construct in Zainal et al. (2025) meta-analysis of 117 DMHI trials
**Modules accessed**	Participant completed ≥1 task within a module (Modules 1–8)	Captures any meaningful engagement with module content beyond login	Equivalent to 'use' construct (Zainal et al., 2025)
**Modules completed (≥80%)**	Participant completed ≥80% of a module's tasks (primary measure)	Captures substantive engagement; 80% chosen for two reasons. First, no participant reached 100% completion on any of the 8 core modules (max observed = 93%), so a 100% threshold would misclassify all engaged participants as non-completers. Second, and more substantively, seven of the ten engaged participants scored between 88-89% on some modules. Under the 90% threshold used in the predecessor trial (Dunn et al., 2024), these participants would have been classified as non-completers on these modules despite having completed almost all available tasks. Therapist session notes indicated that these participants had actively engaged with and applied the content from these modules in their parenting practice, supporting the decision to adopt a more lenient 80% threshold that better reflected their actual level of engagement.	Adapted from Dunn et al. (2024); equivalent to 'completion' construct (Zainal et al., 2025)

Part B: Participant-level engagement data (accessed vs. completed, ≥80%)

Participant ID	Intervention arm	Modules accessed (≥1 task)	Modules completed (≥80%)	Never logged in
**P11**	Self-guided	8	8	No
**P12**	Self-guided	0	0	Yes
**P14**	Self-guided	0	0	Yes
**P16**	Self-guided	0	0	Yes
**P17**	Self-guided	0	0	Yes
**P18**	Self-guided	0	0	Yes
**P20**	Self-guided	1	0	No
**P21**	Self-guided	0	0	Yes
**P22**	Self-guided	0	0	Yes
**P23**	Self-guided	1	1	No
**P24**	Self-guided	0	0	Yes
**P25**	Self-guided	0	0	Yes
**P26**	Self-guided	0	0	Yes
**P27**	Self-guided	0	0	Yes
**P28**	Self-guided	0	0	Yes
**P01**	Therapist-support	4	4	No
**P02**	Therapist-support	7	7	No
**P03**	Therapist-support	0	0	Yes
**P04**	Therapist-support	3	3	No
**P05**	Therapist-support	8	8	No
**P06**	Therapist-support	8	8	No
**P07**	Therapist-support	8	8	No
**P08**	Therapist-support	0	0	Yes
**P09**	Therapist-support	6	6	No
**P10**	Therapist-support	8	8	No
**P13**	Therapist-support	0	0	Yes
**P15**	Therapist-support	0	0	Yes
**P19**	Therapist-support	1	0	No

Part C: Summary statistics

**Measure**	**Self-guided (*n* = 15)**	**Therapist-support (*n* = 13)**	**Overall (*n* = 28)**
**Never logged in, *n* (%)**	12 (80%)	4 (31%)	16 (57%)
**Completed all 8 modules (≥80%), *n* (%)**	1 (7%)	4 (31%)	5 (18%)
**Accessed all 8 modules (≥1 task), *n* (%)**	1 (7%)	4 (31%)	5 (18%)
**Mean modules completed (≥80%), M (SD)**	0.6 (2.1)	4.0 (3.6)	2.2 (3.3)
**Mean modules accessed (≥1 task), M (SD)**	0.7 (2.1)	4.2 (3.7)	2.3 (3.3)

Note. "Accessed" = completed ≥1 task in a module; "Completed" = completed ≥80% of a module's tasks. The two measures produced near-identical results overall. No participant reached 100% completion on any of the 8 core modules (max observed = 93%). Module 1 data combines the starter module (Module 0) and Module 1, taking the higher of the two completion percentages. The predecessor trial (Dunn et al., 2024) used a 90% completion threshold; applying that threshold here would have classified each of the five fully-engaged participants as completing only four of eight modules, hence the more lenient 80% threshold was adopted for this study. As arms were not randomly assigned and differed in recruitment pathway and timing, these comparisons describe two non-equivalent groups (see Section 4.5).

Table S11. Individual SCARED-A (parent anxiety) scores and reliable change index (*n* = 11).

PID	Arm	Modules completed (≥80%)	T1	T2	T3	RCI T1→T2	RCI T1→T3	Reliable change T1→T2	Reliable change T1→T3	Above clinical threshold at T3[2]
**1**	Therapist-support	4	107	87	86	−3.46	−3.63	Reliable improvement	Reliable improvement	Yes
**2**	Therapist-support	7	56	41	47	−2.60	−1.56	Reliable improvement	No reliable change	Yes
**3**	Therapist-support	0	66	94	—	+4.85	—	Reliable deterioration	—	—
**4**	Therapist-support	3	57	68	—	+1.90	—	No reliable change	—	—
**5**	Therapist-support	8	94	100	86	+1.04	−1.38	No reliable change	No reliable change	Yes
**6**	Therapist-support	8	81	71	65	−1.73	−2.77	No reliable change	Reliable improvement	Yes
**7 ***	Therapist-support	8	78	106	88	+4.85	+1.73	Reliable deterioration	No reliable change	Yes
**8**	Therapist-support	0	70	60	73	−1.73	+0.52	No reliable change	No reliable change	Yes
**9**	Therapist-support	6	63	49	40	−2.42	−3.98	Reliable improvement	Reliable improvement	Yes
**10**	Therapist-support	8	51	10	7	−7.10	−7.61	Reliable improvement	Reliable improvement	No
**11**	Self-guided	8	81	86	77	+0.87	−0.69	No reliable change	No reliable change	Yes

[1] *Reliable Change Index (RCI) = (x2 − x1) / SEdiff, where SEdiff = √(2 × (SD × √(1−r))²). |RCI| > 1.96 = reliable change (p < .05); negative = improvement (anxiety ↓), positive = deterioration (anxiety ↑). Constants: SCARED-A normative SD = 16.68, α = .94, SEdiff = 5.78 (Jacobson & Truax, 1991; van Steensel & Bögels, 2014). T1→T2 = baseline to post-intervention (8 weeks); T1→T3 = baseline to six-month follow-up. '—' = no data for that interval.* [2] *Above clinical threshold = SCARED-A score ≥30 (females) / ≥20 (males) at T3 (van Steensel & Bögels, 2014). All 11 participants in this sample were female and the ≥30 threshold was applied throughout.*

Modules completed = number of core modules (0–8) for which the participant completed ≥80% of tasks; 0 = never logged in or no module engagement beyond the starter module.

** P07 showed an increase in parent anxiety scores post-intervention; this is interpreted in clinical context in Section 3.8. At baseline, P07 presented with high anxiety and significant agoraphobic difficulties, requiring volunteer support to attend sessions. The rise in parent anxiety at T2 is understood as reflecting engagement with previously avoided situations (i.e., exposure) rather than deterioration. P07 achieved progress on both personal and module-specific goals and demonstrated increased confidence and functional engagement. Wellbeing was monitored throughout; follow-up discussions with Home-Start at study completion and one year later identified no adverse effects and reported positive feedback from all parents, including P07. P03 showed a reliable increase*

on the parent SCARED-A at T2 (RCI = +4.85); however, P03 never logged into the intervention and did not attend any therapy contacts. This change cannot be attributed to the intervention. Home-Start did not report any adverse effects for P03.

Table S12. Individual SCAS-Preschool (child anxiety, <5 years) scores and reliable change index (*n* = 7).

PID	Arm	Modules completed	T1	T2	T3	RCI T1→T2	RCI T1→T3	Reliable change T1→T2	Reliable change T1→T3
**1**	Therapist-support	4	27	15	30	−1.58	+0.39	No reliable change	No reliable change
**2**	Therapist-support	7	4	14	23	+1.32	+2.50	No reliable change	Reliable deterioration
**5**	Therapist-support	8	53	44	31	−1.18	−2.89	No reliable change	Reliable improvement
**7**	Therapist-support	8	34	66	59	+4.21	+3.29	Reliable deterioration	Reliable deterioration
**8**	Therapist-support	0	84	74	50	−1.32	−4.47	No reliable change	Reliable improvement
**9**	Therapist-support	6	56	46	39	−1.32	−2.24	No reliable change	Reliable improvement
**11**	Self-guided	8	53	52	45	−0.13	−1.05	No reliable change	No reliable change

[1] *RCI formula and interpretation as per Table S11. Constants: SCAS-Preschool normative SD = 19.0, α = .92, SEdiff = 7.60 (Edwards, Rapee, & Kennedy, 2010). Reliable change threshold: |RCI| > 1.96 (p < .05); negative = improvement (anxiety ↓), positive = deterioration (anxiety ↑). T1→T2 = baseline to post-intervention (8 weeks); T1→T3 = baseline to six-month follow-up. '—' = no data for that interval. Modules completed = number of core modules (0–8) for which the participant completed ≥80% of tasks; 0 = never logged in or no module engagement beyond the starter module.*

** PID 7 showed reliable deterioration in child anxiety at both T2 and T3. This parent (P07) engaged with exposure-based content targeting their own agoraphobic difficulties; the increase in child-reported anxiety is hypothesised as reflecting a temporary disruption to household routines and parental availability as the parent engaged with previously avoided situations, consistent with the short-term rebound pattern noted in Section 4.1. Scores show partial recovery by T3. Wellbeing was monitored throughout; Home-Start reported no adverse effects.*

** PID 2 showed reliable deterioration in child anxiety at T3 (RCI = +2.50). The baseline SCAS-Preschool score of 4 is near the floor of the measure (maximum = 56; clinical threshold ≥34), suggesting the T1 score may underrepresent this child's typical anxiety level. The apparent deterioration at T3 is therefore more plausibly attributable to a floor or regression-to-mean effect than to genuine clinical worsening, particularly given that this parent showed concurrent reliable improvement in their own anxiety (SCARED-A T1→41, RCI = −2.60).*

Table S13. Individual SCAS-P (child anxiety, ≥5 years) scores and reliable change index (T2; $n = 4$; T3 $n = 2$).

PID	Arm	Modules completed	T1	T2	T3	RCI T1→T2	RCI T1→T3	Reliable change T1→T2	Reliable change T1→T3
3	Therapist-support	0	69	90	—	+2.86	—	Reliable deterioration	—
4	Therapist-support	3	51	52	—	+0.14	—	No reliable change	—
6	Therapist-support	8	61	69	62	+1.09	+0.14	No reliable change	No reliable change
10	Therapist-support	8	57	51	43	−0.82	−1.91	No reliable change	No reliable change

[1] *RCI formula and interpretation as per Table S11. Constants: SCAS-P normative SD = 16.4, α = 0.90, SEdiff = 7.33 (Brown-Jacobsen, Wallace, & Whiteside, 2011). Reliable change threshold: |RCI| > 1.96 ($p < .05$); negative = improvement (anxiety ↓), positive = deterioration (anxiety ↑). T1→T2 = baseline to post-intervention (8 weeks); T1→T3 = baseline to six-month follow-up. T3 $n = 2$; individual scores (62 and 43) are reported in Table 4 of the main manuscript. '—' = no data for that interval. Modules completed = number of core modules (0–8) for which the participant completed ≥80% of tasks; 0 = never logged in or no module engagement beyond the starter module.*

* *PID 3 showed reliable deterioration in child (SCAS-P) scores at T2 (RCI = +2.86). However, this parent (P03) never logged into the intervention and did not attend any therapy contacts; this change therefore cannot be attributed to the intervention or any exposure process. Home-Start did not report any adverse effects for P03.*
